# Does Participative Leadership Matters in Employees’ Outcomes During COVID-19? Role of Leader Behavioral Integrity

**DOI:** 10.3389/fpsyg.2021.646442

**Published:** 2021-05-21

**Authors:** Muhammad Usman, Usman Ghani, Jin Cheng, Tahir Farid, Sadaf Iqbal

**Affiliations:** ^1^Department of Business Administration, Iqra National University, Peshawar, Pakistan; ^2^School of Management, Xiamen University, Xiamen, China; ^3^Department of Business Administration, Iqra University, Karachi, Pakistan; ^4^School of Education, Zhejiang University, Hangzhou, China; ^5^Department of Applied Psychology and Behavioral Sciences, Zhejiang University, Hangzhou, China; ^6^Department of Psychology, Foundation University Islamabad, Rawalpindi, Pakistan

**Keywords:** participative leadership, leader’s behavioral integrity, workplace thriving, helping behaviors, COVID-19

## Abstract

The coronavirus pandemic (COVID-19) has badly affected the social, physical, and emotional health of workers, especially those working in the healthcare sectors. Drawing on social exchange theory, we investigated the effects of participative leadership on employees’ workplace thriving and helping behaviors among frontline workers during the COVID-19 pandemic. In addition, we examined the moderating role of a leader’s behavioral integrity in strengthening the relationship between participative leadership, and employees’ workplace thriving and helping behaviors. By using a two-wave time-lagged design and data collected from 244 healthcare workers, a moderated hierarchal regression was implemented to test the proposed hypotheses. As hypothesized, participative leadership predicted employees’ workplace thriving and helping behaviors. The leader’s behavioral integrity strengthened the relationship between participative leadership and employees’ thriving and moderated the relationship between participative leadership helping behaviors. Implications for research, theory, and practice are discussed.

## Introduction

The COVID-19 pandemic appears to have been the most significant phenomenon of 2020 and has badly affected the health system worldwide (Ornell et al., 2020). According to the United Nations (2020), this ongoing respiratory disease is the greatest challenge we have faced after the Second World War. As of January 9, 2021, there are over 88.9 million confirmed cases, including 1.9 million deaths. The USA reported over 22 million cases, India 10.4 million, Brazil 8.01 million, Russia 3.32 million, the United Kingdom 2.96 million, France 2.74 million, Turkey 2.30 million, Italy 2.23 million, Spain 2.05 million, and Pakistan about half a million. Until that date, the USA, Brazil, and India had the highest death toll, with 369,390, 201,460, and 150,570, respectively (World Health Organization, 2020).

No single organization has been able to escape from the consequences of the new disease, COVID-19, which has drastically influenced all walks of life, including our social functioning, economy, health, and services (Antonakis, 2020; McKibbin and Fernando, 2020). We have witnessed a substantial workforce reigned by fear, confusion, despair, and uncertainties (Charoensukmongkol and Phungsoonthorn, 2020). Beyond the significant insights from epidemiology, medicine, economics, and psychology, different leadership approaches are also vital to answering the challenges thrown up by this critical disease (Kniffin et al., 2020).

The effect of leadership style is thought to be decisive in a crisis, and its response to such situations could quickly shift organizations’ social, economic, and health status, which ultimately uplifts employees’ well-being (Dirani et al., 2020). Therefore, in this study, an exchange-based model is developed and empirically tested to investigate participative leaders’ impact on healthcare workers’ outcomes, in particular employees’ workplace thriving and helping behaviors while also exploring the moderating effect of a leader’s behavioral integrity. Specifically, we hypothesize that participative leaders can help employees in a crisis-induced work environment to nurture them to thriving and increase their helping behaviors. Importantly, this research aims to contribute to a participative leadership role in the COVID-19 context by exploring two critical issues.

First, as literature on leadership in healthcare settings during crises is scarce (Bartsch et al., 2020), to fill this gap, we examine the role of participative leadership’s effectiveness in predicting employees’ workplace thriving and helping behaviors during the COVID-19 crisis. Participative leadership focuses on shared influence and joint decision-making between leaders and subordinates (Koopman and Wierdsma, 1998), to provide employees with greater discretion, attention, and support and solicit their involvement in addressing problems and making decisions (Nystrom, 1990). In the light of social exchange theory (Blau, 1964; Huang et al., 2010) when leaders treat employees well, they are expected to reciprocate by exhibiting higher work performance and putting extra effort to contribute to the organization (Moorman, 1991). Based on this notion, we posit that healthcare workers are more likely to thrive and extend more helping behaviors toward others in the presence of participative leadership.

Second, to better understand when participative leadership is more effective in nurturing employees’ workplace thriving and their helping behaviors, the current study’s important motivation is to examine its boundary conditions. Considering the limited studies on digital transformation and leadership (Uhl-Bien and Arena, 2018), we examine the moderating role of a leader’s behavioral integrity (LBI) on the relationship between participative leadership and employees’ workplace thriving and helping behaviors.

We choose LBI as a potential boundary condition for three reasons. First, past studies reveal its positive effect on employees’ job outcomes: organizational commitment (Fritz et al., 2013), employees’ performance (Leroy et al., 2012), creativity, and extra-role efforts (Way et al., 2018; Peng and Wei, 2019). Second, earlier studies also show that LBI has many implications for the effectiveness of leaders’ behaviors in shaping desirable job outcomes for employees (Kannan-Narasimhan and Lawrence, 2012; Peng and Wei, 2019). Accordingly, we argue that LBI also has implications for participative leadership’s effectiveness in shaping employees’ workplace thriving and helping behaviors. Finally, prior studies recognized LBI’s relevance in highly demanding business environments (Yang et al., 2019) which can arise due to the crisis of COVID-19. As the COVID-19 pandemic is often referred to as a significant digital transformation driver (Iansiti and Richards, 2020), based on the LBI as a boundary condition, we expect participative leadership to affect healthcare staff’s job outcomes more profoundly.

The structure of the current paper is as follows. The theoretical background and hypotheses development are presented in Theoretical Background and Hypotheses Development section. The methodology including sampling and procedures, measurement, and data analysis are developed in Methodology section. The results are given in Results section. The discussion and implications are provided in Discussion section. Limitations and future directions are offered in Limitations and Future Work Guidelines section. Finally, Conclusion section comprises the conclusion.

## Theoretical Background and Hypotheses Development

### Social Exchange Theory

Social exchange theory (SET: Blau, 1964) is considered one of the most prominent theories in organizational behavior and we have used this for our framework. SET explains that a good deed by an exchange partner (i.e., a leader) engenders the other (i.e., a subordinate) to feel obligated to reciprocate with positive behaviors (Gouldner, 1960). Individuals who observe their leader as a worthy role model are likely to feel obligated to their leadership and exhibit more interest in their assigned work (Liborius, 2014).

The social exchange view has been developed and adjusted in a range of leadership literature (e.g., Eisenberger et al., 2010; Li and Liao, 2014). Since social exchange contains unspecified expectations and obligations of future returns, subordinates react positively to leaders’ favorable conduct based on the norm of reciprocity (Gouldner, 1960; Blau, 1964). According to the SET, when leaders provide employees with decision-making, autonomy, and support, subordinates are more likely to reciprocate with positive attitudes and behaviors to their supervisors (Gouldner, 1960). For example, when leaders have built high-quality associations with their employees, these employees tend to exhibit more thriving at work, OCB, and other favorable outcomes (Organ, 1990; Chan, 2019; Usman et al., 2021). In a similar vein, recent research also reveals that individuals experience higher energy levels when they sense positive exchanges with their supervisors (Atwater and Carmeli, 2009). Relying on the social exchange perspective, we argue that employees who are encouraged by their leaders through participative leadership behaviors, such as involvement in decision-making and being given more responsibility, and so this autonomy may lead individuals to thrive more and offer helping behaviors toward co-workers.

### Participative Leadership and Employees’ Workplace Thriving

Workplace thriving (Spreitzer et al., 2005) refers to a mental state of both “vitality” and “learning” that an individual experiences during his/her work. Prior studies acknowledge vitality and learning as two essential dimensions of workplace thriving. Vitality represents one’s energized feeling and presents as an eagerness for work (Nix et al., 1999). In comparison, learning refers to one’s attainment of knowledge and the application of skills to shape confidence and capability in the work setting (Carver, 1998). Workplace thriving indicates a psychological experience and is subject largely to the peripheral environment’s influence.

Drawing on the self-determination theory, Spreitzer et al. (2005) suggested a socially embedded workplace thriving model. According to Spreitzer et al. (2005) a sound working environment, rich work-related resources, and robust motivational conduct are effective reasons for employees’ workplace thriving. In particular, positive situational features, such as employees’ work environment and how they accomplish their job tasks (including information sharing, decision-making, and the degree of mutual trust during interpersonal communication), are essential for individuals to thrive.

Prior studies show that various positive leadership styles, such as servant leadership (Usman et al., 2021), empowering leadership (Li et al., 2016), ambidextrous leadership (Usman et al., 2020), authentic leadership (Mortier et al., 2016), and transformational leadership (Niessen et al., 2017), are important in fostering positive individual employee outcomes such as workplace thriving. However, although participative leadership leads to several positive outcomes (Bortoluzzi et al., 2014; Chan, 2019; Chang et al., 2019), its impact on workplace thriving is still relatively unexplored in research studies.

In essence, participative leadership affects individuals’ work contexts, provides them with essential working resources, and enhances their motivation levels (Spreitzer, 1995). First, participative leadership encourages employees to be involved in decision-making (Kahai et al., 1997; Somech, 2006) and offers responsibility, power, and autonomy to subordinates (Kirkman and Rosen, 1999). Subsequently, it enables them to have comparatively high decision-making power and self-direction and undertake accurate and timely decisions and actions. Second, this empowering context improves individuals’ active involvement in the work setting and may enhance employees’ working vitality and learning motivations.

Besides, the social learning view on participative leadership posits a learning process by offering individuals more intrinsic motivation and rewards from the work context (Thomas and Velthouse, 1990). This includes encouraging subordinates into the decision-making practice and taking more time to establish progressive interpersonal relations with their subordinates (Kozlowski et al., 1999). Recent research reveals that individuals experience higher energy levels when they sense positive exchanges with their supervisors (Atwater and Carmeli, 2009). We argue that this will, in turn, nurture employees’ workplace thriving because, when individuals know the meaning of their work, they increase attention to their tasks and involvement in their work (Orvis et al., 2009). Therefore, we hypothesize:

*Hypothesis 1*: Participative leadership is significantly positively associated with employees’ workplace thriving.

### Participative Leadership and Helping Behavior

Participative leadership is described as the actions that empower the employees and offer them prospects to be involved in independent decision-making processes. Active involvement in decision-making encourages employees to trust in their leader’s abilities and skills. When individuals gain experience and skills, they get appreciation, and their leader recognizes their novel ideas and skills. This belief in them by their supervisors urges them to carry out additional tasks and responsibilities that enhance the organization’s competitiveness and growth (Lu et al., 2015). A participative leader stimulates motivation *via* involving employees in the decision-making process (Kahai et al., 1997; Somech, 2003); this involvement makes them feel that leaders value their ideas and suggestions. Consequently, the autonomy of sharing ideas and low control of a participative leader intrinsically motivate followers to exhibit more helping behaviors (Sagnak, 2016).

Organ (1990) reveals that the exchange-based (Blau, 1964; Huang et al., 2010) model is particularly relevant in understanding employees’ discretionary behaviors (e.g., helping) in the workplace. If individuals view their exchange as fair, in turn, they will feel an obligation to respond to leaders by exhibiting citizenship behavior (Farh et al., 1990; Organ, 1990). Helping co-workers is a kind of employee citizenship behavior acknowledged to benefit leaders and organizations (Settoon et al., 1996; Masterson et al., 2000). For instance, assisting co-workers with a task at hand, on which his/her leader is dependent, assists in furthering the leader’s goals related to his/her job (Poon, 2006). Likewise, if skillful subordinates offer support in orienting new co-workers, it might facilitate the leadership to “conserve energy” and spend more time on essential aspects of his/her job (Podsakoff and MacKenzie, 1997). In this regard, employees’ helping behaviors toward co-workers are considered a type of reciprocity for valuable resources exchanged in a socio-emotional association with the leadership (Korsgaard et al., 2002; Yang and Mossholder, 2010). For these reasons, we hypothesize the following:

*Hypothesis 2*: Participative leadership is positively related to employees’ helping behaviors.

### Moderating Role of LBI

Simons (2002, p. 19) defines LBI as “the perceived consistency between a leader’s words and deeds.” Prior studies (e.g., Palanski et al., 2011; Leroy et al., 2012; Yang et al., 2019), have empirically and theoretically tested and validated that LBI is a relatively different construct from other leadership traits such as servant leadership, authentic leadership, and ethical leadership. Perceived LBI entails both the perceived promise-keeping and alignment between espoused and enacted values, irrespective of moral principles (Simons, 2002). Even though perceived LBI could include adherence to immoral or anti-social values (Peng and Wei, 2019), in practice, “impression management concerns describe that vast majority of managerially supported values are socially desirable or positive” (Way et al., 2018, p. 766).

Past studies reveal that LBI significantly and positively predicts several work-related outcomes for employees. For example, LBI leads to employees’ organizational commitment (Leroy et al., 2012; Simons et al., 2015), creativity (Peng and Wei, 2019), and in-role and extra-role performances (e.g., Leroy et al., 2012; Way et al., 2018). On this basis, we posit that combining participative leadership with LBI is likely to yield the most beneficial effects on employees’ work-related behaviors during the COVID-19 crisis, as LBI theory suggests that LBI is particularly important in extremely demanding situations (Leroy et al., 2012). On one hand, it will stimulate employees’ intrinsic motivation to put more effort into their work (Fry et al., 2005), thereby fostering employees’ thriving. On the other hand, by establishing the norms of desirable workplace behaviors *via* a consistent pattern of words-deed alignment (Simons, 2002), trust in leaders will develop (Simons et al., 2015). In turn, employees will feel an obligation to reciprocate by exhibiting citizenship behaviors such as helping behaviors (Farh et al., 1990).

In terms of the opposite situation, we contend that individuals who observe their leaders’ integrity as low are less likely to be motivated to involve themselves in participative leadership. This is because, when employees perceive their leaders as low in integrity, they might feel uncertain about what constitutes desirable and appropriate behavioral norms in their workplace (Simons, 2002; Simons et al., 2007), leading to less motivation to show agreement with these leaders’ values (Hewlin et al., 2017). Consequently, employees might less receptive and attentive to their tasks, rendering a low degree of psychological resourcefulness that ultimately suppresses their capacity and enthusiasm to do their work (Yang et al., 2019). As a result, we argue that employees may be less likely to embrace participative leadership and this then weakens the relationship between participative leadership and workplace thriving.

**Figure 1:**
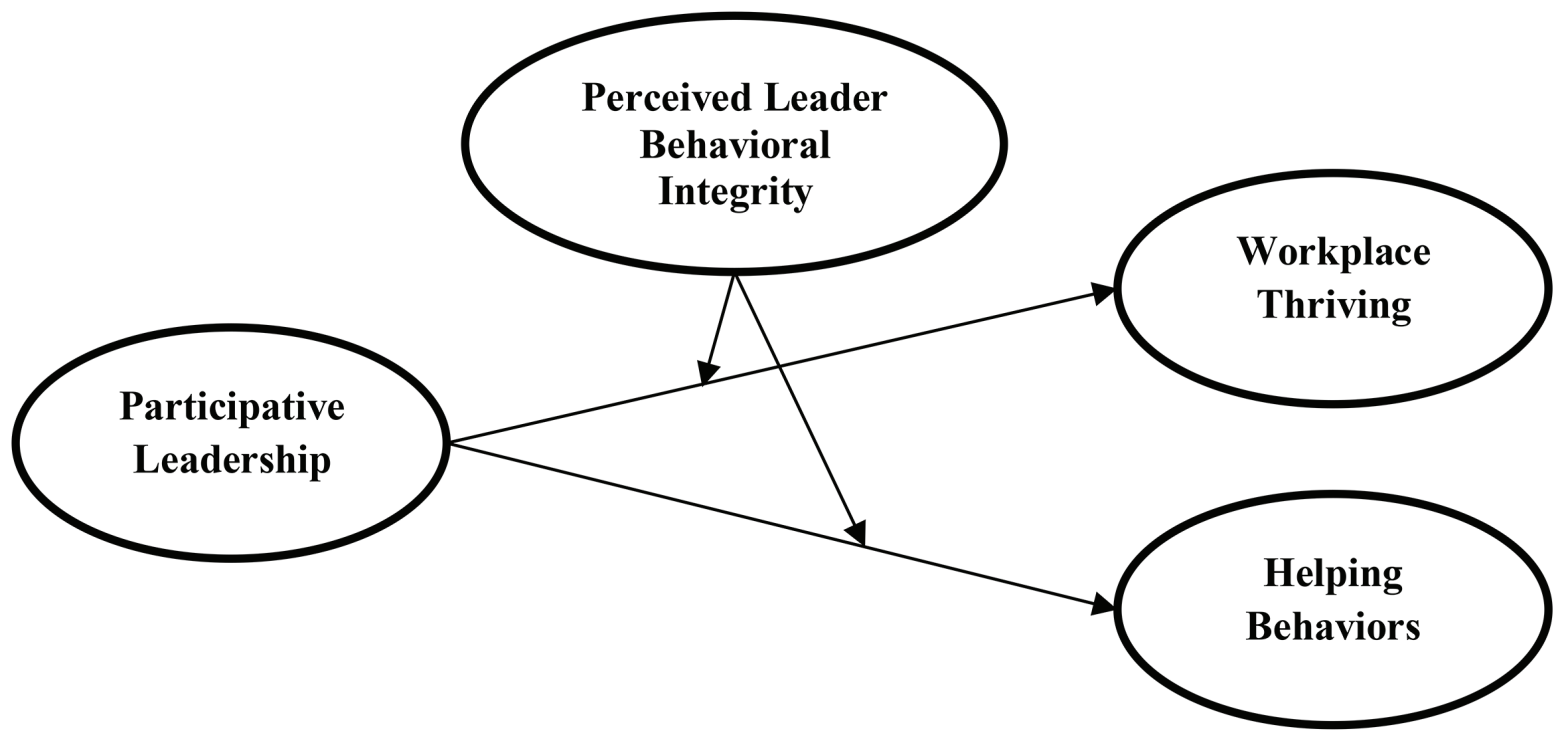
Proposed model.

Moreover, leaders with BI would have a greater inclination to be self-centered and care more about their own benefits than those of their subordinates’ (Jiang et al., 2014). Hence, these leaders are likely to conflict with the value of altruism characteristic of participative leadership. Thus, when individuals consider his/her leaders as lacking in integrity, as a result, they will be less likely to identify with their leaders, which unbalances the association between participative leadership and employee helping behaviors. Likewise, knowing that trustworthiness is a distinctive feature of leaders observed as higher in integrity (Hewlin et al., 2017), individuals with a lower perception of their leaders’ integrity will form low levels of trust with those leaders (Hinkin and Schriesheim, 2015). Subsequently, they may embrace participative leadership less and this weakens the relationship between participative leadership and employee helping behaviors (Figure 1).

*Hypothesis 3a*: Perceived LBI moderates the association between participative leadership and workplace thriving, such that the positive association is stronger for individuals who perceive their leaders as having high LBI rather than low.

*Hypothesis 3b*: Perceived LBI moderates the association between participative leadership and helping behaviors, such that the positive association is stronger for individuals who perceive their leaders as having high LBI rather than low.

## Methodology

### Sampling and Procedures

The current study used a time-lagged design to collect data from the healthcare sector of Khyber Pakhtunkhwa (KPK) Province in Pakistan. Participation was voluntary. The administered questionnaires were distributed and collected within a designated time. Accessibility sampling was used in data collection. All the participants were educated about the purpose of the study, methods, and researcher affiliations. We told the participants that they have the right to refuse or break their commitment to participate any time without any reprisal. We also ensured the confidentiality of the participants’ responses by collecting data anonymously and reporting the aggregated results. The data were collected through two surveys managed 2 months apart. This approach was used to minimize the common method issue, as recommended by Podsakoff et al. (2012). Prior studies, for example, Farid et al. (2019) and Usman et al. (2021), also used this approach. Participative leadership and LBI were measured at Time 1, and employees’ workplace thriving and helping behavior were measured at Time 2.

We collected 244 questionnaires, eight of which were not properly completed and hence removed, leaving a usable sample of 236. This sample included 26.3% males and 73.7% females, 24.2% participants were below 25 years of age, 58.9% were aged 25–33, and 16.9% were above 33, 22.5% had the experience of 1–4 years, 48.7% participants had 4–8, while 28.8% had over 8.

### Measures

#### Participative Leadership

Participative leadership was measured with a six-item scale adapted from a study by Arnold et al. (2000), an example item being “The supervisor encourages us to express our opinions and suggestions.” The Cronbach’s alpha of their study was 0.91. See Table 1 in Validity and Reliability section for the current study’s Cronbach’s alpha values.

**Table 1:** Measurement analysis summary.

Variables	Factor loadings	Cronbach *α*	Composite reliability	AVE
Participative leadership	0.770–899	0.936	0.938	0.717
Perceived leadership behavioral integrity	0.762–949	0.935	0.937	0.715
Workplace thriving	0.702–0.869	0.935	0.936	0.597
Helping behavior	0.701–0.865	0.909	0.911	0.597

#### Perceived Leadership Behavioral Integrity

Perceived leadership behavioral integrity was assessed with a six-item scale adapted from a study by Moorman et al. (2013). An example item is “Leaders in my organization will do what they say.” Their study’s Cronbach’s alpha was 0.91.

#### Workplace Thriving

Workplace thriving was evaluated with a 10-item scale by Porath et al. (2012). Example items include “I see myself continually improving” and “I have energy and spirit” Their Cronbach’s alpha value was 0.92.

#### Helping Behavior

Helping behavior was measured with a seven-item adapted scale (Van Dyne and LePine, 1998) with an example item being “I am used to helping others in their work responsibilities.” The Cronbach’s alpha value they reported for this scale was 0.85.

#### Control Variables

We controlled the demographic variables such as age, gender, and education because previous studies have shown these to have some influence on the study variables (Kanfer and Ackerman, 2004; Uchino et al., 2006; Purvanova and Muros, 2010).

### Data Analysis

A confirmatory factor analysis was conducted to check the measurement model fitness indices such as *χ*^2^, SRMR, RMSEA, and CFI (Hu and Bentler, 1999). Cronbach’s alpha and composite reliability and average variance extracted were calculated to check the constructs’ reliability and validity. To check the common method bias issue, Harman’s single-factor test was performed. Finally, hierarchical linear regression analysis was applied to test the study’s proposed model. We used this technique as it provides step-by-step outcomes of study variables (Ghani et al., 2020b; Nadeem et al., 2020), and has recently been widely employed by researchers (Tariq and Ding, 2018; Ali et al., 2019; Ghani et al., 2020a) as a means to confirm their proposed models.

## Results

### Common Method Bias

We collected data from a single source in the current study; therefore, CMB may be an issue in the data (Podsakoff et al., 2012). Harman’s single-factor test indicated that the most total variance was explained by the first factor (32.68%). As this is less than 50%, CMB is not an issue in the data. Moreover, the inter-correlation of the study variables was less than 0.90, indicating the non-existence of CMB.

### Validity and Reliability

To assess the validity and reliability of the construct, we followed methods used by Fornell and Larcker (1981), Bagozzi and Yi (1988) and Hinkin (1998). Table 1 shows factor loadings above 0.60, Cronbach’s alpha values greater than 0.70, composite reliability values greater than 0.60, and average variance extracted values greater than 0.50, confirming convergent validity. Further, Table 2 exhibits the square root value of AVE higher than the inter-correlation coefficients of the constructs, showing good discriminant validity (Ali et al., 2020; Rasool et al., 2020, 2021).

**Table 2:** Means, SD, and correlations results.

	Mean	SD	1	2	3	4	5	6	7
1.Gender	1.74	0.44	1						
2.Age	1.93	0.64	−0.098	1					
3.Experience	2.06	0.72	−0.014	0.038	1				
4.PL	4.86	1.63	0.170^**^	−0.027	−0.004	**0.847**			
5.PLBI	5.08	1.55	−0.156^*^	−0.014	−0.037	0.232^**^	**0.846**		
6.WT	4.43	1.38	0.053	0.101	0.040	0.414^**^	0.216^**^	**0.772**	
7.HB	4.73	1.41	0.113	0.001	−0.014	0.581^**^	0.274^**^	0.572^**^	**0.772**

*n* = 236, PL, participative leadership; PLBI, perceived leader behavioral integrity; WT, workplace thriving; HB, helping behaviors. The bold values are the square roots of AVE.

* *p* < 0.05;

** *p* < 0.01.

### Measurement Model

After assessing the convergent and discriminant validities, measurement model fitness indices were also evaluated. The measurement model’s fitness achieved the threshold criteria proposed by Hu and Bentler (1999) and Hair et al. (2007), *χ*^2^ = 2.125, SRMR = 0.053, RMSEA = 0.078, and CFI = 0.905, thus all are within range.

Table 2 shows the correlation results among the study variables. The results indicate that all the relationships are in their expected directions.

### Hierarchical Linear Regression Analysis

The hierarchical linear regression was conducted *via* SPSS (see Table 3). Results showed that participative leadership positively and significantly influenced workplace thriving (*β* = 0.418, *p* < 0.01, Model 2), supporting H1. Similarly, participative leadership positively and significantly influence helping behaviors (*β* = 0.579, *p* < 0.01, Model 6), and hence, H2 is also supported. Furthermore, the significant interaction coefficients (*β* = 0.194, *p* < 0.01, Model 4) and (*β* = 0.234, *p* < 0.01, Model 6) confirm the moderation effect of perceived leader behavioral integrity in the relationships between participative leadership with workplace thriving and with helping behaviors, supporting H3a and H3b.

**Table 3:** Hierarchical regression results.

	Workplace thriving				Helping behavior			
	M1	M2	M3	M4	M5	M6	M7	M8
Gender	0.064	−0.006	0.021	−0.010	0.114	0.017	0.049	0.012
Age	0.106	0.111	0.114	0.126^*^	0.012	0.019	0.022	0.037
Experience	0.036	0.037	0.042	0.058	−0.013	−0.013	−0.007	0.012
PL		0.418^**^	0.382^**^	0.384^**^		0.579^**^	0.537^**^	0.539^**^
PLBI			0.133^*^	0.208^**^			0.157^**^	0.248^**^
PL × PLBI				0.194^**^				0.234^**^
R2	0.016	0.185	0.201	0.231	0.013	0.339	0.361	0.405
ΔR2	0.003	0.170	0.016	0.030	0.013	0.326	0.022	0.044
F	0.302	13.129^**^	11.592^**^	11.482^**^	1.031	29.575^**^	25.981^**^	25.942^**^

PL, participative leadership; PLBI, perceived leadership behavioral integrity.

* *p* < 0.05;

** *p* < 0.01.

**Figure 2:**
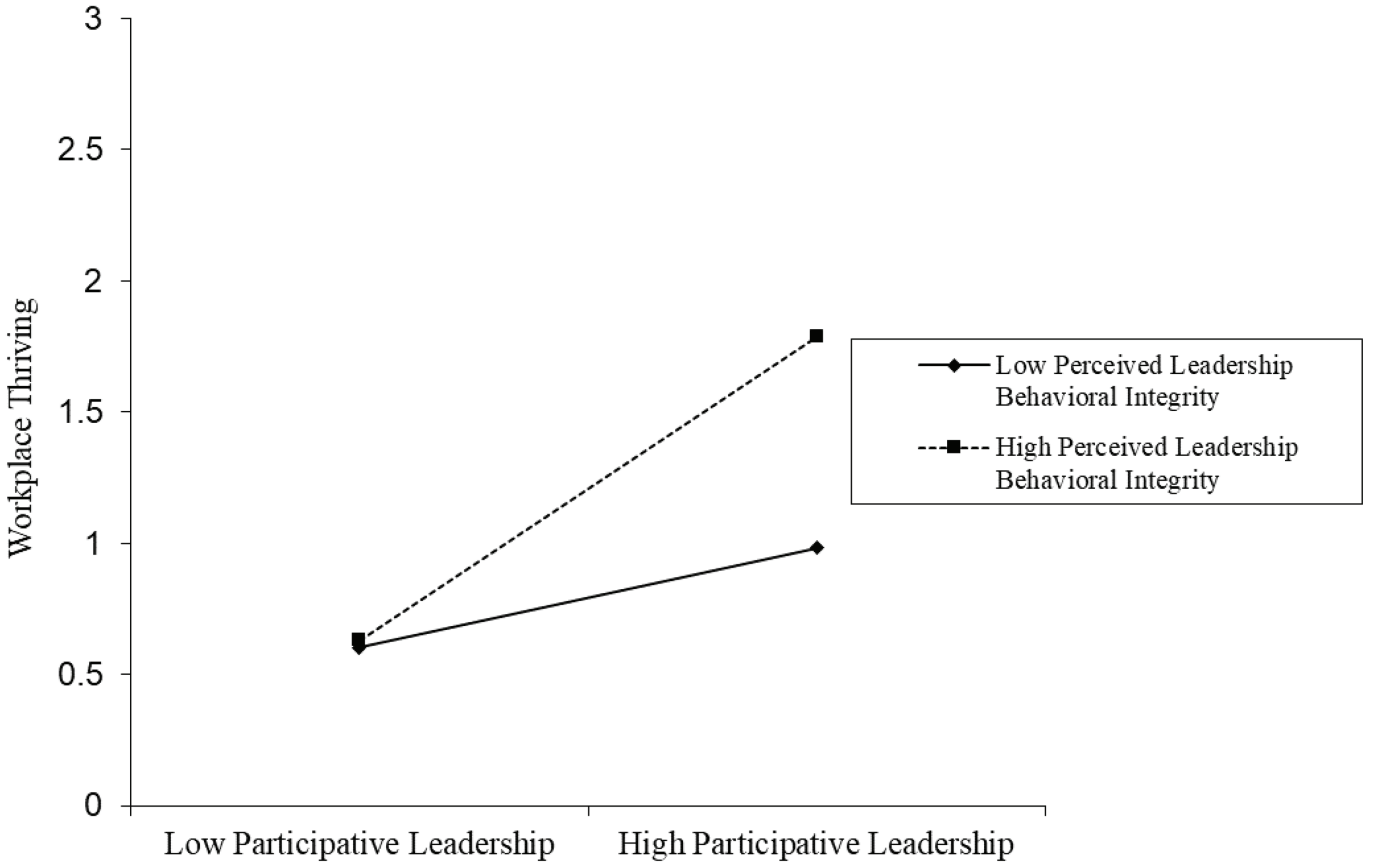
Moderating effect of perceived leadership behavioral integrity between the relationship of participative leadership and workplace thriving.

**Figure 3:**
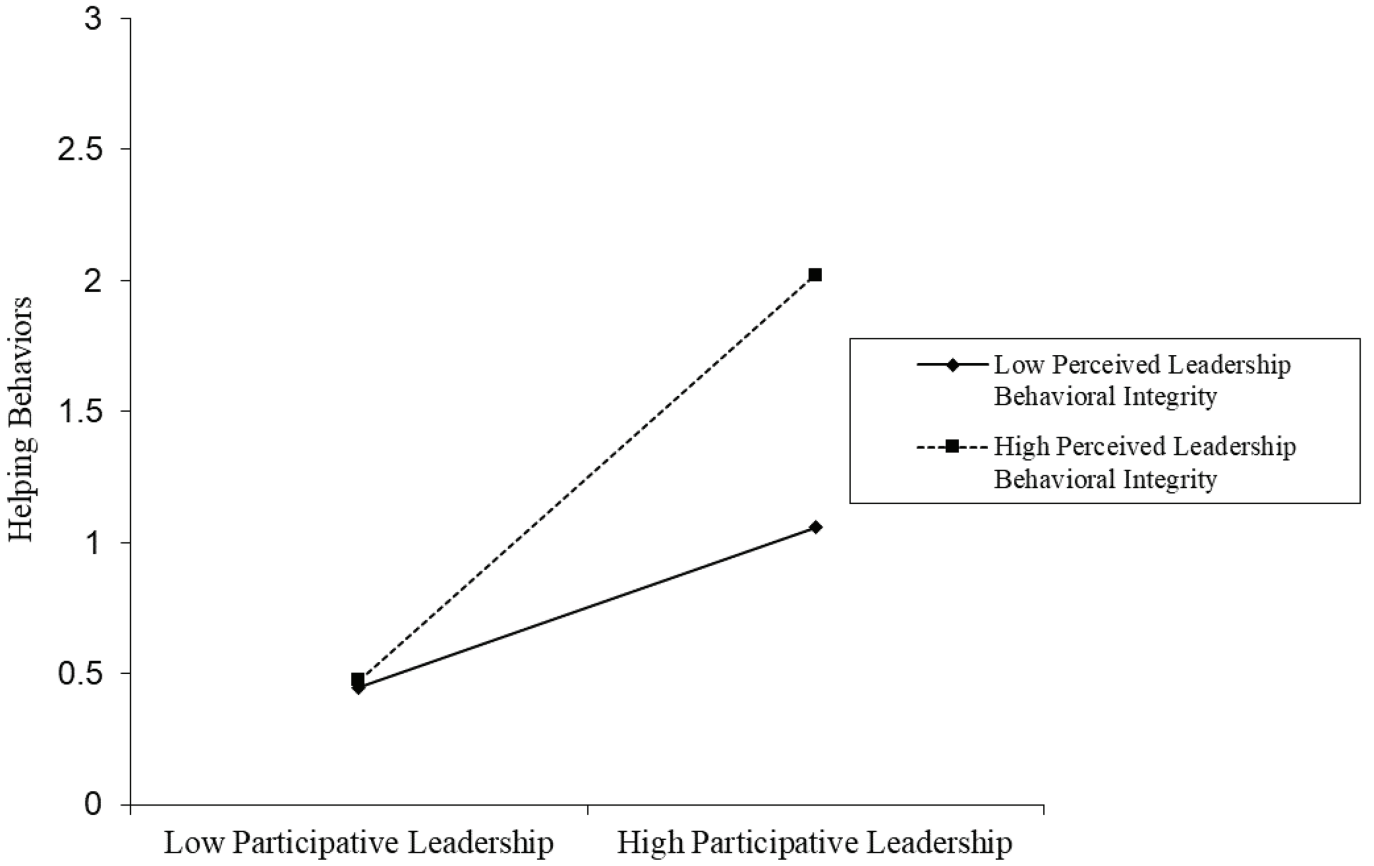
Moderating effect of perceived leadership behavioral integrity between participative leadership and helping behaviors.

The moderating effect of perceived leader behavioral integrity is visualized shown in Figures 2, 3. Further, perceived leader behavioral integrity was split into high (+1 SD) and low (−1 SD) levels to examine the nature of interaction effects. The positive association between participative leadership and workplace thriving is much stronger and more positive (*β* = 0.578, *t* = 8.174, *p* < 0.01) when perceived leader behavioral integrity is high. Unsurprisingly, this relationship is less positive (*β* = 0.190, *t* = 6.008, *p* < 0.01) when such integrity is perceived as low. Similarly, the positive association between participative leadership and helping behavior is much stronger and more positive (*β* = 0.773, *t* = 10.932, *p* < 0.01) when perceived leader behavioral integrity is high and less so (*β* = 0.305, *t* = 9.645, *p* < 0.01) when it is low. These findings provide further support for the moderation hypothesis.

## Discussion

Overall, the current study advances our understanding of participative leadership’s influence by empirically testing its relationship with thriving and helping behaviors and testing how a leader’s integrity moderates this linkage in a novel COVID-19 context. Specifically, using the exchange-based model, we found that participative leadership was positively related to employees’ thriving and helping behaviors during the crisis. Furthermore, findings reveal that the relationship between participative leadership and employees’ work-related outcomes was contingent on the leader’s perceived integrity. Several theoretical and practical implications of the study are discussed below.

### Theoretical Implications

The current study makes three distinct theoretical contributions. First, this study advances the participative leadership literature in the novel COVID-19 context by showing that the participative leadership style nurtures employees’ workplace thriving and helping behaviors. Consistent with prior studies, results show a positive relationship between participative leadership and employees’ workplace thriving. We argued that an important characteristic of participative leadership is embedded in the leader-subordinate relationship, which genuinely gives followers greater discretion, attention, support, and empowerment in decisions (Nystrom, 1990), ultimately enhancing their vitality and learning motivation at work. Second, developing the social exchange perspective, participative leaders’ behaviors stimulate the development of a smooth relationship with followers, which encourages employees’ positive response as helping behaviors. These findings of a positive relationship between participative leadership and work-related outcomes extend the previous empirical findings (Bortoluzzi et al., 2014; Chan, 2019; Chang et al., 2019) from the leadership level to the subordinate level.

Finally, the current study contributes to the effectiveness of participative leadership by empirically testing leader behavioral integrity as an imperative contingent factor for describing the relationship between participative leadership and employees’ work-related outcomes, i.e., workplace thriving and helping behaviors. The findings on the role of perceived LBI (Gatling et al., 2020) are significant and reveal an important theoretical contribution.

Surprisingly, the boundary condition of perceived LBI is not well understood theoretically nor tested empirically. Until now, this has limited our comprehension of the application and usefulness of participative leadership in the work context, particularly in a crisis such as COVID-19. Therefore, this study addresses this research void by developing theoretical reasoning and finding empirical support for our prediction that the association between participative leadership and employees’ thriving and helping behaviors was stronger when LBI was higher and vice versa. This finding shows that LBI has a significant catalyzing influence that amplifies the work outcomes related to participative leadership.

### Practical Implications

The study provides a few important practice implications for healthcare organizations. The findings show that participative leadership is relevant to the healthcare employee context. Participative leaders delegate greater empowerment to their followers and encourage them to actively participate in decision-making, which positively influences their workplace thriving and arouses more helping behaviors. Hence, it is suggested that leaders could reduce an individual’s reluctance and reservations about participating by displaying adequate participative leadership, especially in a crisis (i.e., COVID-19). For example, leaders could extend opportunities and more support for participation, accept their employees’ recommendations, consider a varied range of decision choices, and offer adequate information and resources for subordinates to effectively accomplish the tasks they are participating in.

Building on these findings, we encourage healthcare leaders to learn and use participative leadership behaviors. Furthermore, healthcare organizations are well-advised to take significant steps to facilitate the development of participative leadership behaviors to nurture employees’ thriving and helping behaviors. For example, organizations could initiate advanced leadership development programs (i.e., coaching, mentoring, and structured workshops) where participative leadership behaviors can be learned and stimulated. Moreover, organizations should also pay attention to the leader’s behavioral integrity because this has substantial benefits for supportive leadership, well-being (i.e., thriving), and helping behaviors. A leader’s behavioral integrity can be improved *via* systematic performance management, training, and personal development practices.

## Limitations and Future Work Guidelines

As with all research, the study has certain limitations that may have implications for forthcoming research. First, to test the hypotheses, the current study implemented a time-lagged design with data collected in two surveys administered 2 months apart. Future researchers need to vary the time between two surveys as a means of assessing the extent to which employees’ perceptions of leadership are prolonged. Second, we used the leader’s behavioral integrity as a boundary condition. Scholars may test our theoretical model by exploring additional moderators originating from subordinates’ attributes and dispositional factors that may affect individuals’ reactions to participative leadership. Further, we did not check for any mediating variable. Researchers could search for any possible underlying mechanisms between participative leadership and outcomes, i.e., employees’ thriving at work and helping behaviors. Third, we only collected data from the healthcare system in Pakistan, making it difficult to generalize the results to other organizations and other nations. To enhance its generalizability, research should extend the current findings to other industries.

Fourth, as Pakistan possesses a relationship-oriented culture, it is more likely that our specific cultural context (i.e., a collectivistic culture or high power distance), in which strong cooperation is valued, may have influenced the current study results. Also, there is a significant difference between Pakistani culture and western culture, which may influence employees’ attitudes and behavioral responses (Chen et al., 2020). Future scholars should attempt to replicate our study findings by employing samples from western cultures. Finally, it is expected that global pandemics could happen more frequently in the future (Settele et al., 2020). Therefore, it is important to conduct studies on a large scale to uncover many of the variables that boost employees’ workplace thriving and helping behaviors in response to future epidemics.

## Conclusion

Our study highlights the role of the participative leadership style in linking it with two important employee job outcomes: workplace thriving and helping behaviors. Our results reveal that participative leadership may nurture employees to thrive and boost their helping behaviors, in line with the exchange-based perspective. In addition, the findings underlined the importance of LBI as a potential boundary condition for participative leadership’s effectiveness. We suggest the theoretical understandings gained through this research study will motivate future scholars to explore further how and when participative leader behaviors could enhance employees’ work-related outcomes.

## Data Availability Statement

The raw data supporting the conclusions of this article will be made available by the authors, without undue reservation.

## Ethics Statement

Ethical review and approval was not required for the study on human participants in accordance with the local legislation and institutional requirements. Written informed consent for participation was not required for this study in accordance with the national legislation and the institutional requirements. Written informed consent was not obtained from the individual(s) for the publication of any potentially identifiable images or data included in this article.

## Author Contributions

MU and UG: conceptualization and writing – original draft. UG: formal analysis. TF and SI: methodology. JC: project administration and supervision, writing – review and editing. All authors contributed to the article and approved the submitted version.

### Conflict of Interest

The authors declare that the research was conducted in the absence of any commercial or financial relationships that could be construed as a potential conflict of interest.
